# Synthesis and Characterization of Single-Phase α-Cordierite Glass-Ceramics for LTCC Substrates from Tuff

**DOI:** 10.3390/ma15248758

**Published:** 2022-12-08

**Authors:** Yongsheng Yu, Jinghan Wang, Yuanyuan Yu, Zhaoli Yan, Yanyan Du, Pengfei Chu, Qiangshan Jing, Peng Liu

**Affiliations:** Henan Province Key Laboratory of Utilization of Non-Metallic Mineral in the South of Henan, College of Chemistry and Chemical Engineering, Xinyang Normal University, Xinyang 464000, China

**Keywords:** tuff, α-cordierite, glass-ceramics, LTCC

## Abstract

Single-phase α-cordierite glass-ceramics for a low-temperature co-fired ceramic (LTCC) substrate were fabricated from tuff as the main raw material, using the non-stoichiometric formula of α-cordierite with excess MgO without adding any sintering additives. The sintering/crystallization behavior and the various performances of dielectric properties, thermal expansion, and flexural strength of the glass-ceramics were detected. The results indicated that only single-phase α-cordierite crystal was precipitated from the basic glass sintered at the range 875–950 °C, and μ-cordierite crystal was not observed during the whole sintering-crystallization process. The properties of glass-ceramics were first improved and then deteriorated with the increase in tuff content and sintering temperature. Fortunately, the glass-ceramics sintered at 900 °C with 45 wt.% tuff content possessed excellent properties: high densify (2.62 g∙cm^−3^), applicable flexural strength (136 MPa), low dielectric loss (0.010, at 10 MHz), low dielectric constant (5.12, at 10 MHz, close to α-cordierite), and suitable coefficients of thermal expansion (CTE, 3.89 × 10^−6 ^K^−1^).

## 1. Introduction

With the flying development of microelectronics and information technology, LTCC materials have been widely used in electronic packaging and microwave devices because of their ability to satisfy the requirements of multifunction, integration, high signal propagation speed, and miniaturization [1,2,3,4,5]. In addition, α-cordierite glass-ceramic has been proven to be highly advantageous as an LTCC substrate material because of its unique properties, such as high flexural strength and suitable CTE (close to Si or GaAs), good chemical stability, and low dielectric constant and loss. Furthermore, the glass-ceramics characteristics were also identified properly to be co-fired with highly conductive electrodes, including Au, Ag, and Cu, below 950 °C [6,7,8,9,10].

Currently, there are two crucial stumbling blocks to restrict the development of α-cordierite in the LTCC field. First, the stoichiometric composition of α-cordierite makes it very hard to produce high-density glass-ceramics because of its narrow precipitation temperature range [11]. Nevertheless, the high density is beneficial to obtain high mechanical strength and low dielectric loss. Second, μ-cordierite is preferentially precipitated from the mother glass and then partially or completely transformed to α-cordierite [12,13]. Seriously, the precipitation of other crystalline phases, such as spinel, leucite, and forsterite, make α-cordierite glass-ceramics unsuitable for LTCC applications in the requirement dielectric constant and CTE [14,15]. Therefore, in order to improve the sinterability of cordierite glass powder, there are many measures that can be taken, e.g., adding various sintering aids [16,17,18,19], selecting non-stoichiometric compositions with less Al_2_O_3_ or SiO_2_, or more MgO [13,20,21,22,23,24,25], using nonmetallic minerals (potassium feldspar [15,25,26], kaolin and talc ore [27,28,29,30], and perlite [31]) as raw materials, mainly utilizing the Na_2_O, K_2_O, CaO, and P_2_O_5_ contained in natural minerals as sintering aids, and adjusting the composition of the glass power to directly precipitate α-cordierite during the sintering process [14,26]. Meanwhile, above measures can also reduce the crystallization temperature of the α-cordierite phase. Furthermore, the use of natural minerals as raw materials to prepare α-cordierite glass-ceramics can also reduce the preparation cost and contribute to environmental protection.

This study attempts to prepare α-cordierite glass-ceramics for LTCC substrate by using a natural resource (i.e., tuff) as a raw material, with a non-stoichiometric composition instead of reagent-grade oxides, without adding any sintering aids, which makes the process economical and environmental. Currently, the research of fabricating glass-ceramics for LTCC substrates using cheap tuff mineral has been barely reported. In addition, the glass powder produces α-cordierite directly during sintering in the range 875–950 °C without precipitating μ-cordierite, and the resulting single densified α-cordierite has a lower dielectric loss, perfect dielectric constant (close to α-cordierite), and matched CTE, which are necessary for use as LTCC substrate.

## 2. Experimental Procedure

Shangtianti tuff of Xinyang City, Henan Province was used as the main raw material, and its chemical components are shown in Table 1. The stoichiometry composition of α-cordierite is 2MgO·2Al_2_O_3_·5SiO_2_. α-Cordierite with the chemical formulation 2.6MgO·2Al_2_O_3_·5SiO_2_ was synthesized from the basic glass mainly made up of tuff with a small additive of reagent grade oxides (MgO, Al_2_O_3_, and SiO_2_ were purchased from Sinopharm Chemical Reagent Co., Ltd., Shanghai, China) to compensate for the required formula proportion. The details on the weight percentages of initial raw materials are shown in Table 2. The K_2_O content in MAS40, MAS45, and MAS50 was 5.86 wt.%, 6.59 wt.%, and 7.32 wt.%, respectively.

The ingredients were mixed according to the composition in Table 2 and placed in a corundum crucible, which was then sintered at 1550 °C. The heating and soaking time at 1550 °C was 5 °C/min and 5 h, respectively. The prepared molten material was immersed in deionized water to obtain the basic glass, which was ground in an alumina canister to obtain glass powders with a final average particle size of about 5 μm. Polyvinyl alcohol (5 wt.%) acting as a binder and dry basic glass powder were uniformly mixed, and then pressed to form a Φ 40 mm disc under a uniaxial pressure about 20 MPa. Glass-ceramics were obtained by sintering the prepared green body in an air atmosphere at a specified temperature for 6 h and cooling with the furnace.

The glass transition temperature and crystallization temperature of the glass powder were analyzed by DSC (TA, Q-600, USA) within the 25–1200 °C measuring range under nitrogen atmosphere, and the heating rate to 1200 °C was 10 °C/min. The crystalline phase composition of each sintered sample was analyzed by XRD (Bruker, D8-FOCUS, Ettlingen, Germany). The microstructure of the fresh fracture surface of sintered samples was obtained by FESEM (Hitachi, S-4800, Tokyo, Japan). The density of sintered samples was determined using the drainage method according to the Archimedes principle. CTEs were detected using a dilatometer (Netzsch, DIL-402C, Selb, Germany) from 40 °C to 600 °C in an air atmosphere, and the heating rate was 5 °C/min. The dielectric property was tested using a precision impedance analyzer (Keysight, E4990A, Santa Rosa, CA, USA) on the prepared disc samples with a measurement frequency of 20 Hz to 10 MHz. The flexural strength of samples (30 mm × 5 mm × 2 mm) was determined utilizing the three-point bending method with a cross-head velocity of 4 N/s.

## 3. Results and Discussion

**Figure 1:**
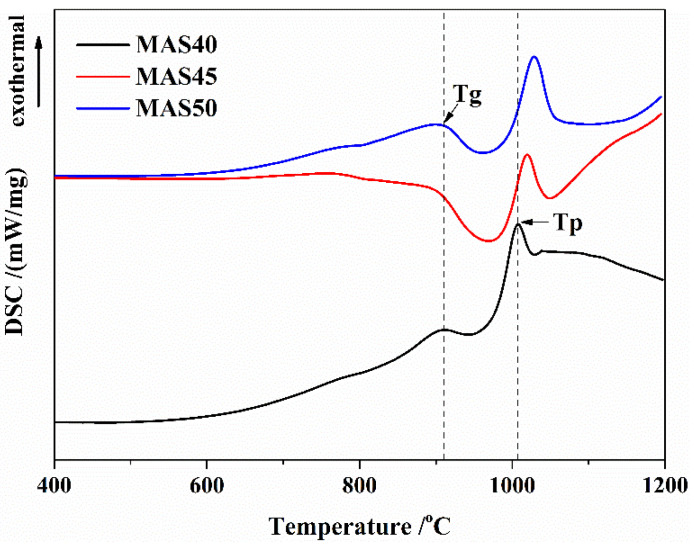
DSC curves of the three parent glasses.

The DSC curves of the three parent glasses were illustrated in Figure 1. The glass transition temperature (Tg) and crystallization peak temperature (Tp) could be obtained from Figure 1, as shown in Table 3. The Tg of the three basic glasses was substantially the same, about 909 °C. Tp was in the range of 1006–1028 °C and lower than that of the formula with kaolin and potassium feldspar as raw material [14,15,26], indicating that the high content of alkaline metal oxide in tuff can reduce the crystallization temperature of basic glass. In addition, there was only one exothermic peak in the DSC curve, illustrating that only one crystalline phase precipitated.

**Figure 2:**
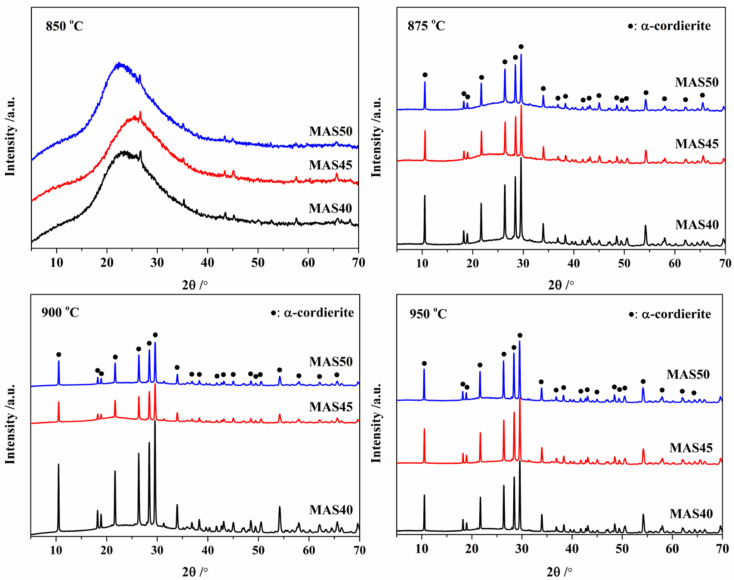
XRD patterns of the glass-ceramics at different sintering temperatures.

The XRD patterns of the glass-ceramic samples prepared in the range 850–950 °C are exhibited in Figure 2. According to Figure 2, no crystal was formed in the sample sintered at 850 °C, but the weak diffraction peak of Al_2_O_3_ at 26.5° was observed. This was because the basic glass was ground in an alumina canister for a long time, causing Al_2_O_3_ to enter into the glass powder, while only α-cordierite phase (PDF: 89–1485) was formed in the samples sintered at 875–950 °C. Normally, excess MgO in cordierite basic glass can lead to forsterite crystallization during the sintering process [32]. Moreover, μ-cordierite always precipitates preferentially from the base glass at low temperatures and then transforms into α-cordierite as the sintering temperature increases [12,13,23,33]. In this study, α-cordierite could be directly precipitated from the basic glass at a low sintering temperature of 875 °C, and only α-cordierite crystal phase was formed. No other crystal phase such as forsterite was formed. The formation of μ-cordierite and the transformation of μ- to α-cordierite was never found, as proven by the DSC and XRD results. This was the same phenomenon as seen for minerals such as potassium feldspar and kaolin [14,26], where alkali metal oxides could break the glass network, reduce the high-temperature viscosity, enhance the particle diffusion, and facilitate the crystallization of α-cordierite, while also inhibiting the formation of μ-cordierite by high potassium content. In addition, α-cordierite was directly generated from the basic glass, which had nothing to do with its particle size and the sintering atmosphere; according to the DSC test atmosphere and Figure 1, this mainly depended on the composition of the basic glass.

**Figure 3:**
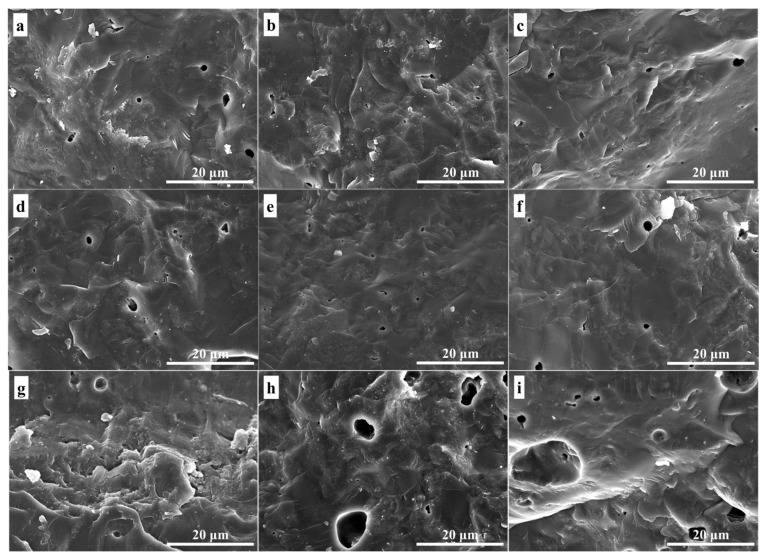
SEM images of fracture of glass-ceramics prepared from MAS40 (**a**,**d**,**g**), MAS45 (**b**,**e**,**h**), and MAS50 (**c**,**f**,**i**) at (**a**–**c**) 875 °, (**d**–**f**) 900 °C, and (**g**–**i**) 950 °C.

**Figure 4:**
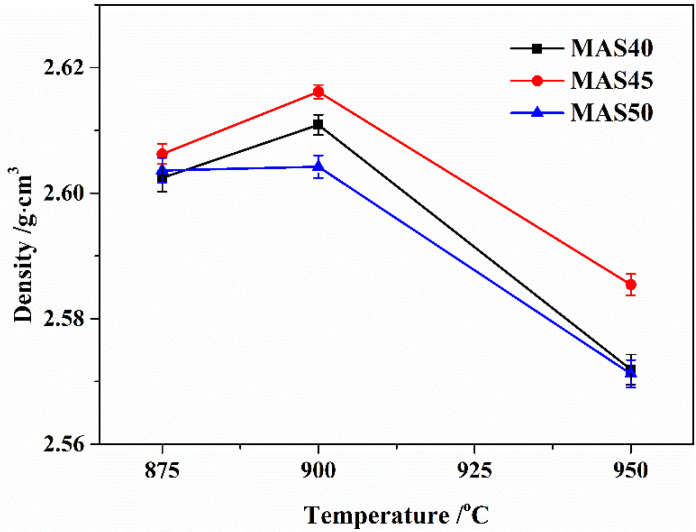
Density of the glass-ceramics sintered at different temperatures.

The microstructure of the fractured surfaces of glass-ceramics samples prepared at 875 °C, 900 °C, and 950 °C is shown in Figure 3. According to Figure 3, the number and size of the closed pores of glass-ceramics decreased first and then increased with the increase in sintering temperature, and there was a similar trend with the amount of tuff. Impurities such as alkali metal oxides and alkaline earth oxides in tuff had the effect of depolymerizing the glass network structure, which could reduce the sintering temperature. Therefore, the low-temperature sintering at 875 °C and 900 °C highly densified the glass-ceramics. However, an excessively high sintering temperature would lead to the basic glass softening rapidly, and the gas inside the glass-ceramics would be unable to discharge and form large closed pores. At the same time, high-temperature sintering could easily induce crystallization pores [34,35], which meant that, during the sinter-crystallizations process, the densification and the crystalline phase formation took place simultaneously, and the crystallization of α-cordierite led to the formation of a new internal porosity. The above factors caused the number and size of pores in glass-ceramics sintered at 950 °C to be higher than at other low-temperature sintered glass-ceramics. This result was consistent with the variation trend of sample density in Figure 4.

The density variation of glass-ceramics sintered in the range 875–950 °C is shown in Figure 4. According to Figure 4, all three basic glasses had excellent sintering properties, and the densities were in the range from 2.57 to 2.62 g∙cm^−3^. The density of glass-ceramics was mainly determined by the chemical composition of the residual glass phase, porosity, and the type and content of crystals. The elements of Mg, Al, Si, and O in the basic glass formed the α-cordierite, and other elements in Table 1 existed in the residual glass phase, including excess Mg, Al, and Si. Generally speaking, crystals always have a more compact structure than the glass phase; hence, the density of glass-ceramics increases with the increase in crystallinity and decreases with the increase in porosity. On the other hand, the increase in sintering temperature and impurity content such as alkali metal oxides can reduce the viscosity of glass, promoting solid-state diffusion and viscous flow, beneficial to the sintering and crystallization process, and finally increasing the density of glass-ceramics. However, when the sintering temperature was too high at 950 °C, more pores were generated (from Figure 3g–i)), reducing the density of glass-ceramics. The above factors led to the lowest density of glass-ceramics prepared at 950 °C, which was still higher than that of glass-ceramics prepared using kaolin [14] and perlite [31].

**Figure 5:**
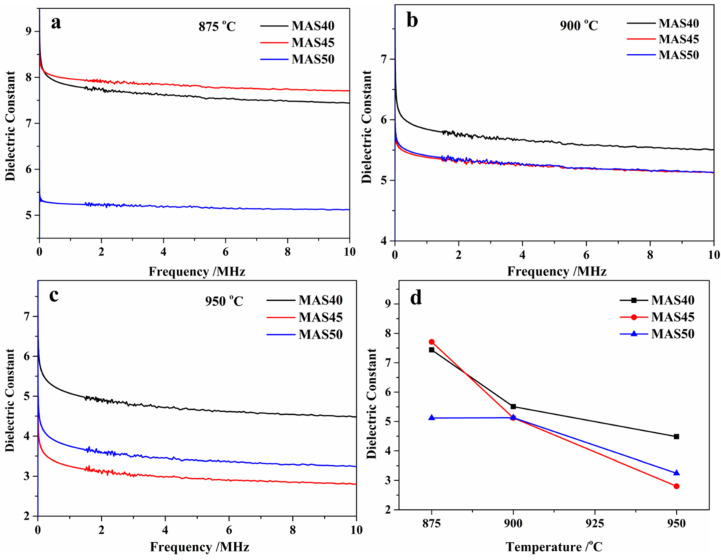
Dielectric constants of glass-ceramics prepared at different sintering temperatures versus frequency (**a**–**c**) in the range 20 Hz–10 MHz and versus temperature (**d**) at 10 MHz.

LTCC substrates and integrated device performance are mainly affected by dielectric properties, including dielectric constant and loss. Multilayer electronic devices require the dielectric constant to be as low as possible because it is a direct function of signal propagation [36]. Dielectric loss is proportional to the ability of electrical energy to be converted into thermal energy. High dielectric loss can result in overheating terminal devices and shortening their service life. Therefore, dielectric loss should be as low as possible. Figure 5 shows the dielectric constants (a, b, and c) of samples prepared in the range 875–950 °C and their dielectric constants (d) at 10 MHz. The dielectric constant of glass-ceramics can be affected by many factors, such as the type and content of the crystalline phase, residual glass phase, porosity, and density [37,38,39]. The dielectric constant of the α-cordierite crystal phase (~5) is smaller than that of the glass phase (~10), and the relative dielectric constant of air is 1. Moreover, the pores in glass-ceramics can drastically reduce the dielectric constant, and this effect is nonlinear. In summary, high crystallinity and high porosity can reduce the dielectric constant of glass-ceramics. Additionally, impurities such as alkali metal oxides remaining in the glass phase of glass-ceramics can intensify the polarization effect, resulting to an increase in dielectric constant.

In this study, crystallinity, porosity, and chemical composition of the residual glass phase were the main factors determining the dielectric constant of glass-ceramics, but their effects were interrelated and sometimes indivisible, and individual contributions could not be distinguished. According to Figure 2, only the samples sintered at 950 °C indicated the absence of an amorphous band between 20°and 30° (2θ), demonstrating that the α-cordierite crystallinity of glass-ceramics sintered at 950 °C was higher than that of other samples. As mentioned before, a higher crystallinity of glass-ceramics results in dielectric constants close to that of α-cordierite. However, the dielectric constants (2.80–4.48) of glass-ceramics sintered at 950 °C were unusually small, indicating that they were mainly affected by the porosity. On the other hand, the dielectric constants (5.12–7.71) of glass-ceramics prepared at 875 °C were mainly dominated by the crystallinity of α-cordierite. Satisfyingly, glass-ceramics sintered at 900 °C had an expected dielectric constant (5.12–5.51) due to its high density and low porosity, which was ideally close to that of α-cordierite glass-ceramics and better than that of some commercial LTCC substrate materials.

**Figure 6:**
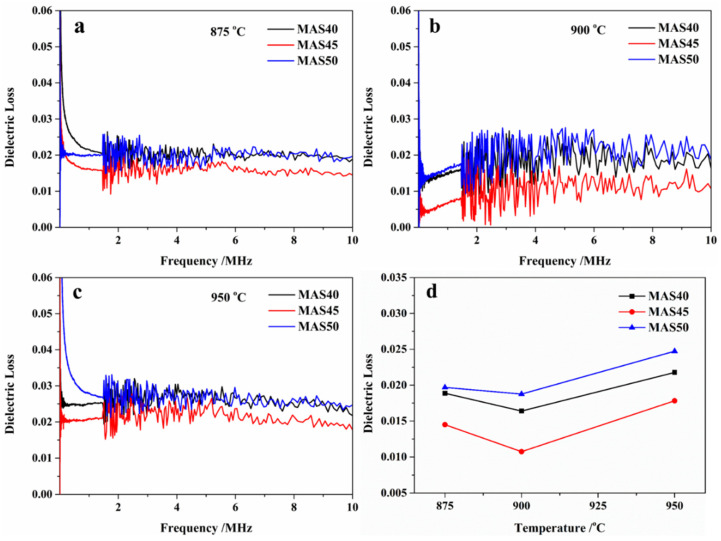
Dielectric losses of glass-ceramics prepared at different sintering temperatures versus frequency (**a**–**c**) in the range 20 Hz–10 MHz and versus temperature (**d**) at 10 MHz.

The dielectric loss comes from intrinsic and extrinsic loss. The intrinsic loss mainly depends on crystal structure, reifying the interaction between the lattice and the external electric field [40,41]. The extrinsic loss is related to the porosity of structural defects, impurities, and micro-cracks of the material’s microstructure [42]. The dielectric loss of a material is mainly affected by extrinsic loss because of the lower intrinsic loss of α-cordierite. Figure 6 shows the dielectric loss (a, b, and c) of glass-ceramics prepared in the range 875–950 °C and its dielectric loss (d) at 10 MHz. According to Figure 6, the dielectric loss of glass-ceramics sintered at 875 °C and 900 °C was between 0.010 and 0.018, while that of glass-ceramics sintered at 950 °C was slightly higher, between 0.017 and 0.24. Due to the presence of impurities in tuff and excess MgO, the dielectric loss was higher than that of cordierite glass-ceramics formed using chemically pure oxides [20,43,44]. Nevertheless, the dielectric losses in this study were still lower than those of glass-ceramics prepared in the published literature [27,28]. The dielectric loss decreased first and then grew with the increase in tuff content, highlighting a similar trend with the increase in sintering temperature. During heat treatment or crystallization, the impurities in tuff raw material did not enter the crystal lattice as interstitials [29], but concentrated in the residual glass phase and accumulated at grain boundaries, resulting in microstructural defects. In addition, metal oxides in tuff raw materials, especially highly conductive metal oxides, can be used as network modifiers to depolymerize the network structure of glass. All the above factors led to an increase in polarization, and finally reduced the increase in dielectric loss of the MAS50 sample. The dielectric loss can be calculated using the formula *P* = *ωεtgδVE*^2^, where *P* is the dielectric loss, *ω* is the phase angle, *ε* is the dielectric constant, *δ* is the tangent of dielectric loss angle, *V* is the volume of the medium, and *E* is the electric field intensity. It can be seen that, when the *ω*, *V*, and *E* are fixed, a smaller *tgδ* or *ε* leads to a lower *P* value. The dielectric constant (~5) and loss (1–2 × 10^−3^) of α-cordierite are smaller than those of the amorphous glass phase. Therefore, a higher crystallinity of α-cordierite leads to a closer dielectric constant of the prepared glass-ceramics to that of α-cordierite, as well as a smaller dielectric loss of the glass-ceramics. In this study, the dielectric loss of low-temperature sintered glass-ceramics was slightly higher, which was mainly caused by the low crystallinity of α-cordierite, while the highest dielectric loss of high-temperature sintered glass-ceramics was mainly due to extrinsic losses such as microstructural defects and high porosity. The trend of dielectric loss of glass-ceramics was similar to that of density; in other words, higher densities led to a lower dielectric loss of glass-ceramic samples with fewer pores.

**Figure 7:**
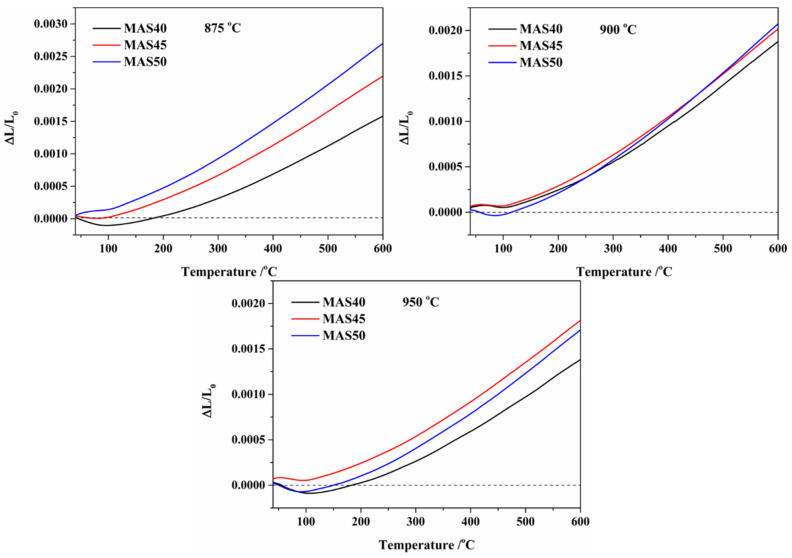
Thermal expansion behavior of the glass-ceramics prepared at different sintering temperatures.

The thermal expansion behavior from 40 °C to 600 °C of samples prepared in the range 875–950 °C is exhibited in Figure 7. By calculating the slopes of the curves of ΔL/L_0_ versus test temperature, the values of the linear CTEs were obtained, as shown in Table 4. From Figure 7 and Table 4, the CTEs of glass-ceramics showed a decreasing trend along with the increase in temperature, but a gradual increase with the amount of tuff. Compared with MgO·Al_2_O_3_·SiO_2_ glass-ceramics prepared at 900 °C by IBM corporation, whose CTE and dielectric constant were 2.5–3.6 × 10^−6^ K^−1^ (room temperature–600 °C) and 5.3–5.7, respectively, these properties were very close to those of the MAS45 sintered at 900 °C in this study. Notably, the slope of the curve obviously transitioned at around 100 °C (MAS40~950 at about 110 °C, MAS45~875 at about 85 °C, and MAS50~900 and 950 at about 90 °C), resulting in slight shrinkage in the range 40–100 °C, and the CTEs of the glass-ceramics were around −2.04–1.56 × 10^−6^ K^−1^. This phenomenon was similar to cordierite glass-ceramics from kaolin, which may have been caused by pores and impurities [14,27]. α-Cordierite possesses extremely low CTE, resulting from its relatively rigid tetrahedral skeleton and the anisotropic expansion of the octahedral skeleton [20,28]. Additionally, α-cordierite’s crystal structure also includes alternate layers of Si_5_Al hexagonal rings linked by Al tetrahedral and Mg octahedral networks. The three-dimensional skeleton can generate two structural holes or vacant sites parallel to the c-axis of a crystal cell [45]. When the vacant sites are occupied by impurities or excess MgO, this results in the expansion of the c-axis [14,45]. Moreover, thermal stress can be induced by this anisotropy, leading to generation of micro-cracks. The above phenomenon was observed in this study, which was manifested in the hysteresis of the thermal expansion curve, leading to a decrease in thermal expansion, which was also observed in the previous literature [14,46]. In addition, the higher porosity in sintered bodies led to the expansion and contraction of α-cordierite crystal. Therefore, the porosity content and micro-cracks in the glass-ceramics may also lower CTEs to a certain extent by absorbing the thermal shock energy [47].

**Figure 8:**
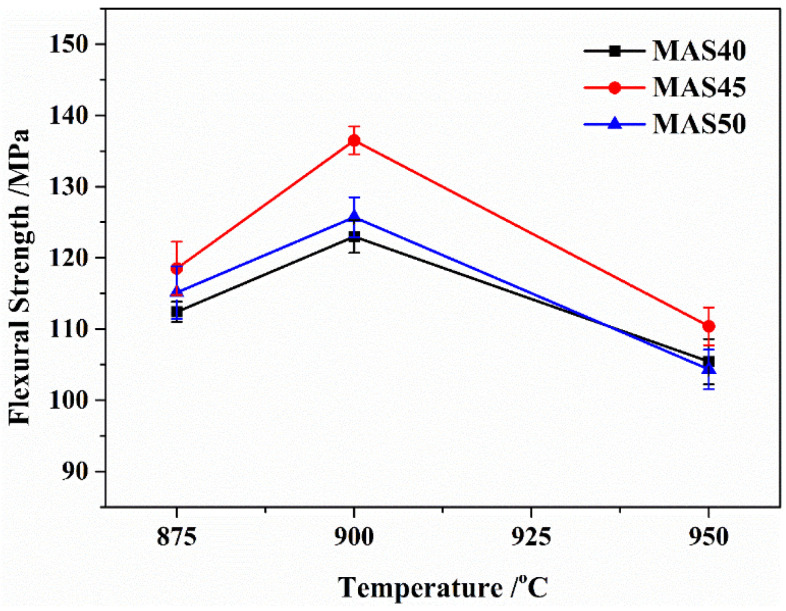
Flexural strength of the glass-ceramics prepared at different sintering temperatures.

The flexural strength of glass-ceramics prepared at temperatures between 875 °C and 950 °C is shown in Figure 8. According to Figure 8, the values of flexural strength were between 104 and 136 MPa, presenting a trend of first increasing and then decreasing with the increase in preparation temperature, as well as the tuff content. This trend was consistent with the density of the samples. In this study, despite the higher crystallinity of α-cordierite (see Figure 2), the glass-ceramics prepared at 950 °C had more pores (see Figure 3) and lower density (see Figure 4), resulting in a lower flexural strength. Meanwhile, the values of flexural strength of the samples prepared at 875 °C were lower due to the lower α-cordierite crystallinity. In other words, the flexural strength of glass-ceramics was mainly affected by the α-cordierite crystallinity at 875 °C and the porosity at 950 °C. These results could be verified by XRD and SEM. High flexural strength is a necessary condition for α-cordierite glass-ceramics used as LTCC substrate materials; hence. electronic integrated devices can exhibit long-term stability in harsh environments. In this study, the glass-ceramics prepared at 900 °C had high flexural strength (123–136 MPa); hence, they can be used as LTCC substrate materials.

## 4. Conclusions

Using tuff as the main raw material, high-density single-phase α-cordierite glass-ceramics were prepared at low temperatures of 875–900 °C without any sintering additives. The content of tuff and sintering temperature were greatly influenced by the sintering/crystallization behavior and properties of glass-ceramics. The impurities from tuff could depolymerize the network structure of the basic glass, which could not only promote the sintering/crystallization behavior of the basic glass, but also adversely deteriorate some properties of the sample. The sintering temperature had a similar effect on the properties of glass-ceramics. Fortunately, the α-cordierite glass-ceramics prepared at 900 °C from the MAS45 formula with a mass fraction of 45 wt.% tuff exhibited excellent comprehensive performance, with high density (2.62 g∙cm^−3^), low dielectric constant (5.12 at 10 MHz) and loss (0.010 at 10 MHz), applicable flexural strength (136 MPa), and suitable CTEs (3.89 × 10^−6^ K^−1^, close to Si). The above properties satisfied the application requirements of LTCC substrate materials; thus, they can act as latent LTCC substrates.

## Figures and Tables

**Table 1:** Chemical compositions of tuff in wt.%.

Compositions	SiO_2_	Al_2_O_3_	MgO	K_2_O	Na_2_O	TiO_2_	CaO	Fe_2_O_3_	Others
Content	69.65	14.34	0.131	12.96	1.73	0.498	0.153	0.261	0.277

**Table 2:** The weight percentages (wt.%) of initial raw materials in the basic glasses.

Samples	MgO	Al_2_O_3_	SiO_2_	Tuff
MAS40	16.00	25.69	18.31	40.00
MAS45	15.84	24.73	14.43	45.00
MAS50	15.75	23.81	10.44	50.00

**Table 3:** Thermal parameters of the three parent glasses.

Basic Glasses	MAS40	MAS45	MAS50
T_g_ (°C)	909.76	908.39	909.43
T_p_ (°C)	1006.22	1020.30	1028.33

**Table 4:** The values of the linear coefficients of thermal expansion.

Content	**Coefficients of Thermal Expansion (CTEs)**								
Temperature Range	40–100 °C (×10^−7^ K^−1^)			100–600 °C (×10^−6^ K^−1^)			40–600 °C (×10^−6^ K^−1^)		
Sintering Temperature	875 °C	900 °C	950 °C	875 °C	900 °C	950 °C	875 °C	900 °C	950 °C
MAS40	−2.04	0.12	−1.73 40–110 °C	3.37	3.65	3.00 110–600 °C	3.37	4.35	5.12
MAS45	−0.76 40–85 °C	0.13	−0.26	4.25 85–600 °C	3.89	3.52	3.65	3.89	4.20
MAS50	1.56	−1.32 40–90 °C	−1.98 40–90 °C	5.12	4.13 90–600 °C	3.49 90–600 °C	2.94	3.52	3.55

## Data Availability

Not applicable.
